# Subacute administration of crude khat (*Catha edulis* F.) extract induces mild to moderate nephrotoxicity in rats

**DOI:** 10.1186/1472-6882-14-66

**Published:** 2014-02-20

**Authors:** Zewdneh Shewamene, Ephrem Engidawork

**Affiliations:** 1 Department of Pharmacology and Clinical Pharmacy, School of Pharmacy, Addis Ababa University, P.O, Box 1176, Addis Ababa, Ethiopia

**Keywords:** Nephrotoxicity, Superoxide dismutase, Catalase, Malondialdehyde, Khat, Rat

## Abstract

**Background:**

Although various studies have been conducted to shed light on the pharmacological actions of khat, little or no data are available regarding khat’s effect on the renal redox system. The aim of this study was therefore to investigate the potential of nephrotoxicity associated with khat exposure in rats.

**Methods:**

Sprague Dawely rats were randomly assigned into eight experimental groups. Animals were treated with Tween80, gentamicin 100 mg/kg and khat at various doses (100, 200 and 400 mg/kg) alone or in combination with gentamicin for ten days. The animals were then sacrificed to obtain blood and renal tissues for subsequent analysis. Renal markers, including creatinine, blood urea nitrogen, antioxidant enzymes as well as markers for lipid peroxidation were determined using established protocols. In addition, histopathological changes were evaluated with hematoxilin and-eosin staining technique.

**Results:**

Lower and moderate doses of khat did not alter the measured parameters compared to controls. By contrast, higher dose (400 mg/kg) of khat not only increased levels of serum creatinine and blood urea nitrogen (p < 0.001) but also levels of malondialdehyde (p < 0.01). Moreover, 400 mg/kg of khat significantly decreased enzymatic activities of superoxide dismutase (p < 0.01) and catalase (p < 0.001). When khat was administered with gentamicin, it was again the higher dose that significantly accentuated gentamicin-induced alterations in the renal system.

**Conclusions:**

Khat treatment at high dose is demonstrated to induce mild to moderate renal damage. Moreover, it creates synergy when combined with nephrotoxic drugs such as gentamicin.

## Background

Khat (*Catha edulis* Forsk) is a shrub or small to medium-sized evergreen tree that belongs to the Celastraceae family. It is cultivated mainly in Yemen and East African Countries [1]. The shrub grows to a height of 6 meters and the leaves are leathery, glossy, brownish green, with serrated edges, arranged in an alternate fashion on straight branches. The young shoots and leaves are parts that are chewed for their psychoactive properties [2].

In Ethiopia, a number of local brands are available, including Aweday, Beleche, Abo mismar, Gelemso and Wondo. It is claimed that the Aweday variety cultivated in Harar highlands of Eastern Ethiopia is the most potent and expensive among the local brands, and hence chosen for export [3,4] as well as for purpose of the present study. Central nervous system stimulation such as euphoria and alertness induced by cathinone, the main active constituent derived from khat chewing, makes it popular among large segments of the society. In addition, factors like easy transportation from village to city khat markets and affordability are thought to play an important role in widening its social use in society. People also believe that khat helps to work more effectively, particularly with manual work, due to increased energy and alertness [5].

Consumption of crude khat extract or its alkaloid fraction preceding stress has been shown to produce oxidative stress in rats by altering activities of serum antioxidant enzymes [6]. Nephrotoxic and hepatotoxic effects are also reported following khat administration to New Zealand white rabbits [7,8]. The generation of free radicals is seriously implicated in khat toxicity following the observation that oral exposure of rats to khat was associated with decreased serum free radicals metabolizing enzymes such as superoxide dismutase (SOD) and catalase [9]. In addition, khat treated rats displayed hepatic enlargement, abnormal findings in serum aspartate aminotransferase, and alkaline phosphatase in both sexes as well as alterations in serum albumin and creatinine in female rats [10].

In spite of the vast published data on the pharmacology and chemistry of khat, toxicological studies with laboratory animals as well as toxicity reports in humans, particularly on the renal system, are scanty. Moreover, the effect of khat-induced redox changes, at least, in the kidney, has not yet been explored. Thus, there is a need for conducting laboratory studies to generate a sufficient body of knowledge in the area. This study therefore attempted to investigate whether exposure of rats to khat had a potential to cause nephrotoxicity via alteration of the renal redox system.

## Methods

### Collection of plant material

Bundles of fresh khat shoots and small branches were purchased (2000 g) fresh at a local market, Aweday, located 525 km South East of Addis Ababa, Ethiopia. The fresh bundles were packed in plastic bags and transported in an icebox to the laboratory. The plant was identified by a taxonomist and a voucher specimen (ZS001) was deposited at the National Herbarium, College of Natural Sciences, Addis Ababa University for future reference. The fresh leaves were immediately kept at -20°C for two days before subjected to extraction.

### Experimental animals

Sixty four (32 male and 32 female) healthy Sprague Dawley rats (6–8 weeks of age and 170 – 210 g) bred in the animal house of School of Pharmacy, Addis Ababa University were used for the experiment. The rats were housed in polypropylene cages and maintained under room temperature (22–25°C), relative humidity of 50% and 12 h light/dark cycle. Animals were provided with pelletized feed and tap water *ad libitum.* All animals were handled according to internationally accepted guidelines [11] and the protocol was approved by the School of Pharmacy Ethics Committee.

### Extraction of khat

Extraction was performed as described elsewhere with slight modification [12,13]. The freeze-dried plant was finely minced, weighed and placed in Erlenmeyer flasks (400 g per flask) wrapped with aluminum foil to avoid light induced decomposition. Chloroform (150 mL) and diethyl ether (450 mL) (1: 3 v/v) were added to cover the minced leaves. The resulting mixture was shaken under dark condition for 24 h using a rotary shaker (New Brunswick Scientific Co, USA) at 120 rpm and 20°C.

The mixture was later filtered through a folded filter paper. The filtrate was again passed through a round filter paper with the help of a mini filter pump. The organic filtrate collected in this way was pooled together in a wide mouth amber bottle and placed in a hood for 24 h to remove the organic solvents. The residue was left overnight in a deep freezer and then lyophilized using a freeze dryer (Christ 100400, Bioblock Scientific, France). The yield was calculated and found to be 1.02%, which was similar with previous works [14-16].

### Grouping and dosing of animals

Animals were randomly assigned into 8 experimental groups, consisting of 8 (four male and four female) animals per group. The first group served as control (CON) and given the vehicle used for reconstitution of the extract (Tween 80, 2% v/v in water). The second, third and fourth groups received crude khat extract at doses of 100 mg/kg (K100), 200 mg/kg (K200) and 400 mg/kg (K400) for ten days. The fifth group (GEN) was treated with gentamicin for eight days at a dose of 100 mg/kg [17]. The rest of the groups received crude khat extract at doses of 100 mg/kg (GK100), 200 mg/kg (GK200) and 400 mg/kg (GK400) for two days before and eight days concomitantly with gentamicin (100 mg/kg). Vehicle and extracts were administered via the oral route, while gentamicin was administered intraperitoneally. Rats were weighed on alternate days and the last known weight was used for dose calculation. The dose for the khat extract was selected based on previous reports [13,14,16].

### Sample collection

Twenty-four hours after the last treatment, animals were slightly anesthetized with ether inhalation and bilateral prilumbal vertical incisions were made to draw blood via cardiac puncture. The blood samples were left at room temperature for 30 min to coagulate. Serum was then separated by centrifuging the samples for 15 min at 3000 rpm and 4°C (Centurion Scientific Ltd K240R, UK). The serum was stored at -20°C for 48 h until subjected to analysis for determination of creatinine and blood urea nitrogen (BUN) levels. In parallel, both kidneys were removed and used for enzyme assay and histopathological studies. The right kidney was rinsed with chilled saline, decapsulated, blotted on a filter paper and quickly weighed. It was then homogenized in ice-cold saline to produce a 10% (w/v) tissue homogenate and stored at -20°C until assayed for the redox markers.

### Biochemical analysis

#### ***Serum creatinine and BUN measurement***

The concentration of serum creatinine and BUN were measured by Cobas integra 400 (Roche, Switzerland) using commercial kits (Roche-Cobas, Switzerland) according to the manufacturer’s protocol. Creatinine level was determined by Jaffe’s reaction without deproteinization, where the samples were subjected to react with picrate in alkaline pH forming a yellow-red color with maximum absorbance at 512 nm. For measurement of BUN level, kinetic test with urease and glutamate dehydrogenase was used. Urea in the sample was hydrolyzed by urease forming ammonia that in turn reacts with 2-oxoglutarate in the presence of glutamate dehydrogenase and reduced nicotinamide adenine dinucleotide (NADH) to produce L-glutamate. The rate of decrease in the NADH concentration is directly proportional to the urea in the sample and this can be determined by measuring the absorbance at 340 nm. The BUN was calculated from urea using a formula BUN (mg/dl) = urea × 0.467.

#### ***Determination of total superoxide dismutase activity***

The total SOD activity was determined using commercial kits (Nanjing NianChen Bioengineering Institute, China). The reaction system consisted of xanthine and xanthine oxidase that produces superoxide radical (O_2_^.-^). The O_2_^.-^ oxidizes hydroxylamine forming nitrite, which colors amaranth by the color developer and this can be assayed at 550 nm (Unic model 2100 spectrophotometer). During the assay, 50 μl of 10% tissue homogenates were mixed well with the reaction system on a vortex mixer (Labnet S0100-230 V, Labnet International Inc., USA) and incubated in a water bath maintained at 37°C (Oakton Stable Temp WD-1250-15, USA) for 40 min. The formation of O_2_^.-^ and nitrite was inhibited by SOD in the samples, reducing the intensity of the amaranth color as well as the absorbance upon addition of the color developing agent. The total SOD activity in the sample was calculated and expressed as U/mg protein. One unit of SOD activity is defined as the amount of SOD that will produce 50% inhibition of oxidation of hydroxylamine induced by xanthine and xanthine oxidase at 37°C in 1 mg/ml protein concentration of tissue homogenate.

#### ***Determination of catalase activity***

Catalase activity was measured based on the manufacturer’s protocol (Nanjing NianChen Bioengineering Institute, China) that relies on the reaction of enzyme in the presence of an optimal concentration of H_2_O_2_. The rate of dismutation of H_2_O_2_ to H_2_O and O_2_ is proportional to the concentration of catalase. Briefly, 50 μl of 10% renal tissue homogenates were mixed well with a known concentration of H_2_O_2_ on a vortex mixer and incubated in a water bath at 37°C for 1 min. Ammonium molybdate was added to the mixture to quench the reaction and react with the remained H_2_O_2_, forming a stable colored complex. The absorbance of the complex was measured at 405 nm. Finally, the catalase catalytic activity of the tissue samples was calculated and expressed as U/mg protein. One unit of catalase catalytic activity is defined as the amount of enzyme that will decompose 1 μmol H_2_O_2_ per second at 37°C in 1 mg protein of tissue homogenate.

#### ***Determination of malondialdehyde level***

The amount of lipid peroxides was calculated as thiobarbituric acid reacting substances such as malondialdehyde (MDA) formed from the breakdown of polyunsaturated fatty acids, which is considered as an index for the peroxidation reaction. The level of MDA in renal homogenate was assayed using commercial kits (Nanjing NianChen Bioengineering Institute, China) based on thiobarbituric acid method, where MDA undergoes condensation reaction with thiobarbituric acid, generating a red product that has a maximum absorption peak at 532 nm. Briefly, tissue homogenates (10%) were well mixed with thiobarbituric acid reaction system in test tubes on a vortex mixer. The test tubes were then sealed with aluminum foil with a hole stung with a needle. The mixture was incubated at 95°C in a water bath for 40 min, cooled with flowing water and then centrifuged at 4000 rpm for 10 min. The supernatant was carefully pippeted into quartz cuvete (Exactaoptech, Germany) to read the absorbance of the red color at 532 nm and MDA level was determined.

### Morphometric analysis

#### ***Body and kidney weight changes***

Body weight of all animals before and after the experiment was taken and the difference was expressed as body weight change. The final day body weight was used for the calculation of body weight change and expression of normalized kidney weight [18]. The weight of both right and left kidneys of each rat was measured at the end of treatment after sacrificing the animal. For standardization, total weight of both kidneys/100 g body weights was determined [19].

#### ***Histopathological examination***

The left kidney tissues fixed in 10% formalin were dehydrated with absolute ethanol solution, cleaned in xylene and embedded in paraffin. The prepared tissue was then sectioned at 5 μm and stained with hematoxylin and eosin for microscopical examination. Coded slides were examined by a blinded pathologist for histopathological changes such as tubular necrosis, inflammation, hyaline casts, and hydropic degeneration.

### Statistical analysis

All data are presented as mean ± standard error of the mean and SPSS data analysis software version 19 was used for data processing. The analysis was performed by one way ANOVA followed by Tukey’s multiple comparison tests. Level of significance was set at p < 0.05.

## Results

### Serum creatinine and blood urea nitrogen levels

Table 1 shows the change in serum markers of all experimental groups. K100 and K200 did not produce detectable changes in the serum markers. Administration of K400, on the other hand, significantly increased creatinine (54.2%, p < 0.001) as well as BUN (30.2%, p < 0.001) levels compared to CON rats. Moreover, K400 rats had a significantly greater levels of the markers compared to K100 (p < 0.001) as well as K200 (p < 0.01 for creatinine and p < 0.05 for BUN) rats. It is of note that levels of both markers were significantly elevated (p < 0.001) in GEN rats compared to all other groups.

**Table 1:** Effects of khat extract on serum creatinine and blood urea nitrogen levels

**Groups**	**Serum creatinine (mg/dl)**	**Blood urea nitrogen (mg/dl)**
**CON**	0.59 ± 0.02	19.92 ± 0.34
**K100**	0.63 ± 0.02	20.15 ± 0.42
**K200**	0.66 ± 0.05	20.79 ± 0.40
**K400**	0.91 ± 0.02^a3b3c2^	25.94 ± 0.53^a3b3c1^
**GEN**	1.24 ± 0.03^a3b3c3d3^	48.25 ± 0.97^a3b3c3d3^

Values are mean ± SEM. n = 8; ^a^compared to CON; ^b^compared to K100; ^c^compared to K200; ^d^compared to K400. ^1^p < 0.05; ^2^p < 0.01; ^3^p < 0.001. (CON: control, received Tween80; K100, khat 100 mg/kg; K200, khat 200 mg/kg; K400, khat 400 mg/kg; GEN, gentamicin 100 mg/kg).

In parallel experiment, khat was administered concomitantly with gentamicin to see whether khat synergizes the toxic effect of gentamicin. The results indicated that whilst no apparent changes were observed with GK100 and GK200, GK400 demonstrated a significantly elevated creatinine (25%, p < 0.001) and BUN (19.9%, p < 0.001) levels compared to GEN rats (Table 2).

**Table 2:** Effects of khat and gentamicin co-administration on serum creatinine and blood urea nitrogen levels

**Groups**	**Serum creatinine (mg/dl)**	**Blood urea nitrogen (mg/dl)**
GEN	1.24 ± 0.03	48.25 ± 0.97
GK100	1.28 ± 0.03	49.42 ± 1.02
GK200	1.30 ± 0.02	49.94 ± 0.71
GK400	1.55 ± 0.06^a3^	57.83 ± 0.79 ^a3^

Values are mean ± SEM. n = 8; ^a^compared to GEN. ^1^p < 0.05; ^2^p < 0.01; ^3^p < 0.001. (GEN: gentamicin 100 mg/kg; GK100: gentamicin 100 mg/kg + Khat 100 mg/kg; GK200: gentamicin 100 mg/kg + Khat 200 mg/kg; GK400: gentamicin 100 mg/kg + Khat 400 mg/kg).

### Effects on antioxidant enzymes and lipid peroxidation

K400 rats exhibited a significant decrease in SOD (14.8%, p < 0.01) and catalase (35%, p < 0.001) activities compared to CON rats. Significantly greater reduction in the activity of both enzymes (p < 0.001 in both cases) was also noted in GEN compared to CON as well as khat-treated rats (Table 3). MDA levels were markedly increased by K200 (41.5%, p < 0.05), K400 (103.8%, p < 0.01) and GEN (141.2%, p < 0.001) compared to CON rats. The increase observed in MDA levels by GEN once again was significantly higher than K100 (119.2%, p < 0.001), K200 (70.3%, p < 0.001) and K400 (18.3%, p < 0.01) rats (Table 3).

**Table 3:** Effects of khat extract on activity of renal antioxidant enzymes and levels of malondialdehyde in rats

**Groups**	**SOD (U/mg protein)**	**CAT (U/mg protein)**	**MDA (nmol/mg protein)**
**CON**	259.50 ± 7.56	17.69 ± 0.73	2.60 ± 0.04
**K100**	249.49 ± 11.49	17.62 ± 0.56	2.86 ± 0.07
**K200**	240.66 ± 6.97	16.20 ± 0.40	3.68 ± 0.17^a1^
**K400**	221.17 ± 7.51^a2^	11.50 ± 0.87^a3^	5.30 ± 0.16^a2^
**GEN**	136.43 ± 4.94^a3b3c3d3^	6.54 ± 0.47 ^a3b3c3d3^	6.27 ± 0.19^a3b3c3d3^

Values are mean ± SEM. n = 8; ^a^compared to CON; ^b^compared to K100; ^c^compared to K200; ^d^compared to K400. ^1^p < 0.05; ^2^p < 0.01; ^3^p < 0.001. (CON: control, received Tween80; K100, khat 100 mg/kg; K200, khat 200 mg/kg; K400, khat 400 mg/kg; GEN, gentamicin 100 mg/kg; SOD, superoxide dismutase; CAT, catalase; MDA, malondialdehyde).

Table 4 illustrates the effect of crude khat extract when given concomitantly with gentamicin on renal redox markers. Compared to GEN rats, GK100 and GK200 tended to have a reduced renal activity of SOD and catalase that failed to reach statistical significance. By contrast, GK400 group revealed a significant reduction in activity of both SOD (25.7%, p < 0.05) and catalase (49.4%, p < 0.01). Whilst no apparent difference was observed with the other doses, khat at a dose of 400 mg/kg along with gentamicin displayed a significant increase (38.6%, p < 0.001) in MDA levels when compared with gentamicin alone.

**Table 4:** Effects of khat and gentamicin co-administration on activity of renal antioxidant enzymes and levels of malondialdehyde in rats

**Groups**	**SOD (U/mg protein)**	**CAT (U/mg protein)**	**MDA (nmol/mg protein)**
GEN	136.43 ± 4.94	6.54 ± 0.47	6.273 ± 0.1974
GK100	134.86 ± 3.23	5.72 ± 0.27	6.749 ± 0.1981
GK200	126.08 ± 8.23	4.60 ± 0.18	6.935 ± 0.2460
GK400	101.33 ± 3.48^a3^	3.31 ± 0.46^a3^	8.694 ± 0.2184^a3^

Values are mean ± SEM. n = 8; ^a^compared to GEN; ^3^p < 0.001. (GEN: gentamicin 100 mg/kg; GK100: gentamicin 100 mg/kg + Khat 100 mg/kg; GK200: gentamicin 100 mg/kg + Khat 200 mg/kg; GK400: gentamicin 100 mg/kg + Khat 400 mg/kg; SOD, superoxide dismutase; CAT, catalase; MDA, malondialdehyde).

### Effects on body weight change and normalized kidney weight

At the end of the experiment, percent body weight change was determined for each group of animals (Tables 5 and 6). It was found out that whereas no appreciable change was observed in body weight change with K100; significant body weight loss was detected with K200 (p < 0.05), K400 (p < 0.01) and GEN (p < 0.001) rats compared to CON. Moreover, body weight loss following gentamicin treatment was significantly greater (p < 0.001) compared to all groups of khat treated rats.

**Table 5:** Effects of khat extract on body weight change and normalized kidney weight

**Groups**	**Body weight change (%)**	**Kidney weight (gm)/100 g body weight**
**CON**	2.83 ± 0.48	0.70 ± 0.01
**K100**	2.22 ± 0.69	0.73 ± 0.01
**K200**	0.32 ± 0.46^a1^	0.74 ± 0.01
**K400**	-4.96 ± 0.28^a2^	0.82 ± 0.01 ^a1^
**GEN**	-10.36 ± 57 ^a3b3c3d3^	0.89 ± 0.01^a3b3c3d3^

Values are mean ± SEM. n = 8; ^a^compared to CON, ^b^compared to K100, ^c^compared to K200, ^d^compared to K400. ^1^p < 0.05; ^2^p < 0.01; ^3^p < 0.001. (CON: control; K100: khat 100 mg/kg; K200: khat 200 mg/kg; K400: khat 400 mg/kg; GEN: gentamicin 100 mg/kg).

**Table 6:** Effects of khat extract and gentamicin co-administration on body weight change and normalized kidney weight

**Groups**	**Body weight change (gm)**	**Kidney weight (gm)/100 g body weight**
GEN	-10.36 ± 0.57	0.89 ± 0.01
GK100	-10.85 ± 0.86	0.89 ± 0.02
GK200	-11.75 ± 0.84	0.90 ± 0.01
GK400	-15.13 ± 1.37 ^a3^	1.00 ± 0.02 ^a2^

Values are mean ± SEM. n = 8; ^a^compared to GEN. ^1^p < 0.05; ^2^p < 0.01; ^3^p < 0.001. (GEN: gentamicin 100 mg/kg; GK100: gentamicin 100 mg/kg + Khat 100 mg/kg; GK200: gentamicin 100 mg/kg + Khat 200 mg/kg; GK400: gentamicin 100 mg/kg + Khat 400 mg/kg).

Rats treated with K100 and K200 showed slight kidney weight gain without statistical significance as compared to CON rats (Table 5). Treatment with crude khat extract at a dose of 400 mg/kg produced a significant (p < 0.05) increase in normalized kidney weight compared to CON. GEN rats also revealed a significantly increased kidney weight gain (p < 0.001) not only compared to CON but also with all khat groups. Significant body weight loss or kidney weight gain was not observed between K100, K200 and K400 rats.

As illustrated in Table 6, concomitant treatment of khat at a dose of 400 mg/kg with gentamicin induced a significantly greater (p < 0.01) body weight loss as well as kidney weight gain (p < 0.01) compared to gentamicin alone.

### Histopathological studies

**Figure 1:**
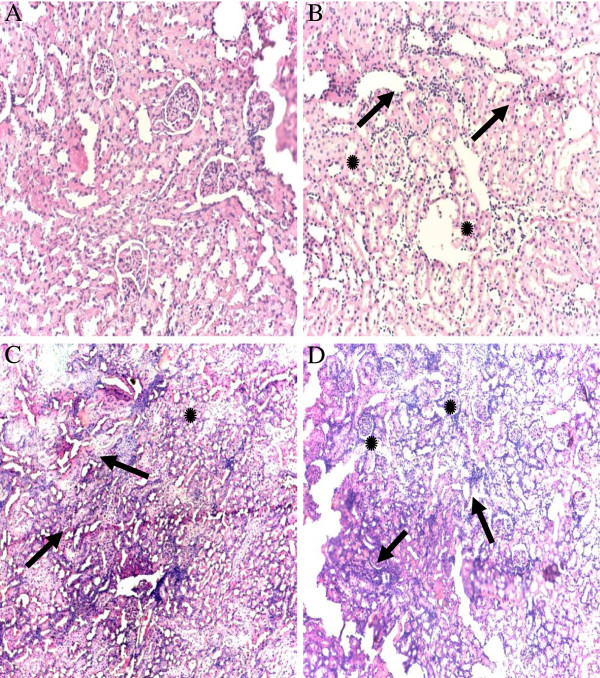
**Photomicrographs of hematoxilin and eiosin stained renal tissues for khat and/or gentamicin treated rats:** The kidneys of the control rats (treated with Tween80) showed normal renal parenchyma with normal histo-architecture **(A)**. On the other hand K400 rats showed mild to moderate interstitial inflammation (arrows), hypertrophied glomerular capillaries and injured dilated Bowman’s capsule (*) **(B)**. GEN rats showed marked infiltrative inflammatory cells and vascular congestion (arrows) and vacuolar degeneration of the tubules (*) **(C)**. Furthermore, GK400 rats revealed more extensive and marked infiltrative inflammation, complete destruction of glomerular capillaries (arrows), degeneration of the tubules and Foamy appearance in the tubular epithelial cells (*) **(D)**. Magnification × 40. K400, khat 400 mg/kg, GEN, gentamicin 100 mg/kg, GK400, khat 400 mg/kg + gentamicin 100 mg/kg.

Histological changes in the kidneys are depicted in Figure 1. Kidneys of control group showed normal renal parenchyma with normal histoarchitecture (Figure 1A). K400 induced histopathological changes as evidenced by mild renal interstitial inflammation, hypertrophied glomerular capillaries and injured dilated Bowman’s capsule (Figure 1B). Gentamicin induced changes were more extensive than khat groups (Figure 1C). Furthermore, GK400 rats revealed more extensive and marked infiltrative inflammation, complete destruction of glomerular capillaries, degeneration of the tubules and foamy appearance in the tubular epithelial cells (Figure 1D).

## Discussion

In this study, the effect of khat administration alone or with gentamicin was studied in terms of alteration of renal markers, including creatinine, BUN, antioxidant enzymes as well as markers for lipid peroxidation. In addition, histopathological changes such as presence of inflammation, tubular degeneration, hyaline casts and vascular congestions were evaluated. Gentamicin-induced nephrotoxicity in rodents is a well documented model for acute renal failure and provides an opportunity to test different compounds or extracts which could have renoprotective properties [20]. Although both sexes of rats were used in this study, there was no apparent difference between males and females in the measured parameters.

### Biochemical changes

Serum creatinine and BUN are commonly used to assess glomeruli filtration rate as well as concentrating and diluting capacity of tubular functions of the kidneys. An increase in values of these markers may indicate development and extent of renal tubular damage [21]. Increased BUN may be associated with kidney disease or failure/blockade of the urinary tract by a kidney stone, congestive heart failure, dehydration or bleeding in the digestive tract [22].

In the present study, administration of khat at a high dose (400 mg/kg) had significantly increased serum creatinine and BUN levels, suggesting that khat use may impair renal function by reducing the ability of kidneys to handle these products. These effects perhaps may originate from changes in the renal blood flow and glomerular filtration rate induced by khat treatment [23].

Al- Motarreb and Broadley [24] reported that khat chewers experience an increase in heart rate and body temperature as well as sweating and cold extremities, which dictate the presence of peripheral vasoconstriction. On the bladder, khat chewing produced a fall in urinary flow rate, an effect that has been shown to be inhibited by the selective α_1_-adrenoceptor antagonist, indoramine, and therefore attributed to activation of this receptor subtype [25]. Peripheral vasoconstriction following khat administration as described above would explain the raised serum creatinine and BUN levels. Compared to khat, gentamicin treatment resulted in a marked elevation of both creatinine and BUN levels. Thus, it is plausible to assume that alteration of these renal indices caused by khat treatment is relatively mild to moderate compared to gentamicin. The fact that the same dose of khat produced significant alteration compared to gentamicin when given along the renotoxic drug reinforces the notion that high dose of khat per se has a direct renotoxic potential.

SOD and catalase are the predominant, if not exclusive, defenses against free radicals in which SOD catalyze the dismutation of superoxide radicals to hydrogen peroxide that in turn is removed by catalase or glutathione peroxidase [26]. In agreement with previous observations [27], the present study also indicated gentamicin-induced oxidative stress, as shown by a significant decrease in kidney catalase and SOD activities. Exhaustion of enzymatic renal oxidative defense mechanisms along with enhanced reactive oxygen species generation could result in oxidative damage in gentamicin treated rats [28]. K400 significantly decreased the activity of both renal SOD and catalase enzymes, suggesting that the extract is able to generate free radicals or directly inhibit the action of these antioxidant enzymes. This finding is concordant with recent studies in which administration of khat extract or its alkaloid fractions were shown to alter activities of the free-radical metabolizing/scavenging enzyme system [6,29].

The peroxidation of lipids gives rise to a number of secondary products, MDA being the principal and most studied one. This aldehyde is a highly toxic molecule and has been considered as more than just a marker of lipid peroxidation [30]. The rationale for MDA as a biomarker relies on the fact that it is solely derived from lipid peroxides and changes in MDA concentration reflects changes in lipid oxidation level [31]. Baliga et al. [32] documented that gentamicin caused lipid peroxidation in the kidney via reactive oxygen species generation. The results in the present study clearly indicated that oral administration of khat at doses of 200 mg/kg and 400 mg/kg had shown accelerated lipid peroxidation in the renal tissues as reflected by an increase in MDA levels, possibly by inducing generation of reactive oxygen species. Indeed, Al-Hashem et al. [29] reported that the toxic effect of khat extract on hepatic and renal functions might be related to lipid peroxidation as indicated by a significant increase in lipid peroxidation biomarkers.

It is, however, worth noting that whilst gentamicin induced elevation of renal MDA levels were accentuated by K400 co-treatment, no apparent change was observed with concomitant administration of K200 and GEN. Moreover, K200 did not reduce antioxidant enzymes to a significant level, though it elevated the MDA levels significantly. This could possibly point to the fact that khat should be able to decrease antioxidant defense mechanisms as well as enhance lipid peroxidation in order to produce significant nephrotoxic effect. This effect appears to be a function of dose, where moderate doses produce lipid peroxidation, probably via increase in free radical production, but high doses are associated with both peroxidation and abrogation of the enzymatic defense mechanism.

Khat is known to contain several constituents, including alkaloids and flavonoids. The flavonoid fraction has been demonstrated to have no effect on antioxidant enzymes activity [6,9], which could probably implicate the alkaloids or other constituents for the observed effect. However, it is widely accepted that antioxidant substances may potentially have deleterious effects (pro-oxidation action), particularly when precise modulation of ROS levels are necessary for normal cell function. The fact that antioxidants may exhibit pro-oxidant activity depending on dosage and presence of free transition metals at cellular sites [33-35], and appearance of the toxicity with higher dose does not totally exclude the contribution of flavonoids.

### Morphologic pathology

K200 and K400 rats showed a significant body weight loss compared to controls, although the extent of loss was lower than gentamicin. Gentamicin-induced weight loss could be associated with direct renal tubular injury. Injury of the renal tubules leads to subsequent loss of tubular cells that take part in renal water reabsorption. This is accompanied by loss of water, leading to dehydration and loss of body weight [36]. On the other hand, increased catabolism associated with gentamicin-induced acute renal failure causes acidosis. Acidosis results in anorexia that in turn decreases oral food intake, eventually culminating in body weight loss [37]. Khat-associated reduction in body weight could therefore be attributed to kidney damage, as it was shown to cause mild-to-moderate injury in the histopathologic evaluation, but it could also be ascribed to khat-induced delay in intestinal absorption that contributes to some degree of malnutrition [38-40] or increased plasma leptin level that leads to loss of appetite [41]. Kidney weight gain probably explained by the edema that was caused by gentamicin and khat induced acute tubular necrosis [19].

The histopathological results were paralleled by serum, antioxidant and lipid peroxidation findings. Rats treated with gentamicin revealed extensive and marked renal tubular necrosis. Although khat alone was not able to produce extensive kidney damage, it resulted in a wide-ranging damage that even included the epithelial cells when combined with gentamicin. This histopathological evidence once again clearly reaffirms the direct nephrotoxic potential of khat. Although the mechanism by which khat produces nephrotoxicity is not clearly known, it is thought to result from local decrease in blood supply, possibly from narrowing of the renal arteries [24,42].

## Conclusions

Administration of khat at higher dose (400 mg/kg) was shown to cause renal damage. Moreover, gentamicin-induced disturbance in renal indices were considerably accentuated by high dose of crude khat extract. Khat, alone or with gentamicin was also found to alter renal histopathology, normalized kidney weight and body weight of rats. Thus, the data collectively indicate that khat does not play a permissive role but has a direct nephrotoxic potential, albeit to a small extent, that creates synergism when combined with other nephrotoxic agents such as gentamicin.

The use of khat at higher dose may cause oxidative stress by depleting anti-oxidative mechanisms or by enhancing pro-oxidant components of tissues, leading to renal injury. More reasonably, khat seems to be able to perturb the delicate balance between protective and damaging mechanisms of a cell that is required for optimal activity, thereby producing oxidative damage.

## Competing interests

The authors declare that they have no competing interest.

## Authors’ contributions

All authors involved in the design and write up of the study, and ZS conducted the actual study and the statistical analysis. Both authors approved the submitted version of the manuscript.

## Pre-publication history

The pre-publication history for this paper can be accessed here:

http://www.biomedcentral.com/1472-6882/14/66/prepub
